# Isolation and characterization of the *EgWRI1* promoter from oil palm (*Elaeis guineensis* Jacq.) and its response to environmental stress and ethylene

**DOI:** 10.1371/journal.pone.0225115

**Published:** 2019-12-04

**Authors:** Qing Zhang, Ruhao Sun, Yusheng Zheng, Yijun Yuan, Dongdong Li

**Affiliations:** 1 Department of Biotechnology, College of Tropical Crops, Hainan University, Hainan, China; 2 Department of Bioengineering, College of Life Sciences and Pharmacy, Hainan University, Haikou, Hainan, China; Huazhong University of Science and Technology, CHINA

## Abstract

WRI1 is a plant-specific transcription factor that enhances the accumulation of oils through the upregulation of the expression of genes involved in glycolysis and fatty acid biosynthesis. In this study, the *EgWRI1* promoter from oil palm was isolated and characterized in transgenic Arabidopsis. The sequence analysis results revealed that various putative plant regulatory elements are present in the *EgWRI1* promoter region. The *EgWRI1* promoter and beta-glucuronidase (GUS) reporter gene were transcriptionally fused and transformed into *Arabidopsis thaliana*. Histochemical analysis revealed that GUS staining was very strong in whole seedlings, especially the stems, leaves, and siliques. Moreover, GUS staining was strong in the silique coats but weak in the seeds. Furthermore, to detect whether *EgWRI1* was induced by environmental stress, we detected the expression efficiency of the *EgWRI1* promoter in transgenic Arabidopsis treated with low temperature, darkness, and exogenous ethylene. The results showed that the activity of the *EgWRI1* promoter was induced by darkness but suppressed significantly when exposed to exogenous ethylene. When treated with low temperature, the activity of the *EgWRI1* promoter was first reduced after 24 hours but recovered after 48 hours. Taken together, these results reveal the features of the *EgWRI1* promoter from oil palm, which will be helpful for improving oil accumulation in oil palm via reasonable cultivation methods.

## Introduction

Triacylglycerols (TAGs) are the most abundant energy-dense storage compounds that accumulate in most plant seeds or fruits. These oils not only provide energy for germination and seedling development before photosynthesis but are also important resources for food and industry applications in daily human life. During the past three decades, substantial progress has been made in oil biosynthesis and its related regulatory network in plants. In seeds and fruits, sucrose is the common energy source for the synthesis of starch, storage proteins, and oils [1]. There are three main steps in oil biosynthesis: glycolysis in both the cytosol and plastid, fatty acid synthesis in the plastid, and TAG assembly in the endoplasmic reticulum (ER). Many previous studies of plants have indicated that the transcription factor WRI1 enhances oil accumulation through the upregulation of the expression of genes involved in both the late stages of glycolysis and fatty acid biosynthesis but not TAG assembly [2–5].

WRI1 is a plant-specific transcription factor of the APETALA2 (AP2)/ethylene-responsive element-binding protein family with two AP2 DNA-binding domains [6,7]. Both LEAFY COTYLEDON (LEC) 1 and 2 function as positive regulators upstream of *WRI1* and specify the regulatory action towards fatty acid metabolism [8]. The Arabidopsis *wri1* mutant exhibits an 80% reduction in oil in seeds and a wrinkled appearance [6,9]. However, the overexpression of *WRI1* leads to an increase in the oil content in both seeds and vegetative tissues [4,10–12].

Oil palm (*Elaeis guineensis* Jacq.) is the highest oil yielding plant in the world, accounting for approximately 40% of the total vegetable oil production worldwide [3,13,14]. The transcriptomic study of oil palm fruits at different developmental stages indicated that the upregulation of genes involved in late glycolysis and fatty acid synthesis are controlled by the transcription factor EgWRI1 [3,15]. Furthermore, EgWRI1-1 restored the low-oil-content phenotype of the Arabidopsis *wri1* mutant and activated the expression of oil biosynthesis pathway genes [16–18]. Additionally, the overexpression of *EgWRI1* in *Nicotiana benthamiana* leaves induced a severalfold increase in leaf TAG levels [19]. However, unlike Arabidopsis, seed maturation master regulators, such as LEC1, LEC2, FUSCA3 and ABSCISIC ACID INSENSITIVE3, are lacking in oil palm [3,8]. Recently, three ABA-responsive transcription factors (EgNF-YA3, EgNF-YC2 and EgABI5) were identified from oil palm and confirmed to activate oil accumulation by directly regulating the expression of *EgWRI1* and genes involved in oil biosynthesis [17]. Nonetheless, seed development in general is governed not only by a complex network of transcription factors that integrate external cues, e.g., light, but also by internal signals, such as the concentration of the plant growth factor [6].

In this study, the promoter of *EgWRI1* from oil palm was isolated and characterized. Putative regulatory elements of the *EgWRI1* promoter were first predicted by PlantCARE. The expression pattern mediated by the *EgWRI1* promoter in transgenic Arabidopsis was also analyzed by the GUS histochemical assay. To detect whether *EgWRI1* can be induced by low temperature, darkness, or phytohormones, the efficiency of the *EgWRI1* promoter in transgenic Arabidopsis separately treated with low temperature, darkness, and ethylene was measured. These results revealed the features of the *EgWRI1* promoter and changes in EgWRI1 activity under different growth conditions.

## Materials and methods

### Plant materials and growth conditions

Oil palm materials were collected from the coconut research institute, Chinese agricultural academy of tropical crops, Wenchang, Hainan, China. Wild-type *Arabidopsis thaliana* ecotype Columbia was used in this study. Arabidopsis plants were cultivated in a growth chamber at 23°C with a 16 h photoperiod (16 h of 150 μE m^-2^ sec^-1^ light and 8 h of darkness).

### Cloning of EgWRI1 promoter and bioinformatic analysis

The promoter region of *EgWRI1* was chosen from the sequence 5’ upstream of translation start site ATG. Putative functional promoter elements were predicted by the PlantCARE (Plant *cis*-acting regulatory elements) database (http://bioinformatics.psb.ugent.be/webtools/plantcare/html/) [20]. The predicted promoter region of *EgWRI1* was cloned from genomic DNA of oil palm leaves by PCR amplification and the specific primers were EgWRI1-proF: 5’- AGAAGCTTGACTGCAATATATGCTTTCAA -3’ (*Hind*III) and EgWRI1-proR: 5’- TAGGTACCGTTCTAGAAAGGGATTATTTCCT -3’ (*Kpn*I). Reactions were performed with Q5 High-Fidelity DNA Polymerase (New England Biolabs) under the following PCR conditions: an initial denaturation at 98°C for 30s, 30 cycles at of 98°C for 10 s, 58.8°C for 30s, 72°C for 60 s and a final extension step of 72°C for 5 min. Then, the PCR product was purified and cloned into the pEASY-Blunt vector (TransGen biotech, Beijing, China) and sequenced.

### Plant vector construction and transformation of Arabidopsis

The fragments of *EgWRI1* promoter were digested with *Hind*III and *Kpn*I from pEASY vector, and subcloned into the multiple cloning site of the pCAMBIA 1300S-GUS vector by replacing CaMV35S promoter. The resulting construct, designated as ProEgWRI1::GUS, was transformed into *Agrobacterium* strain GV3101 by electroporation [21], and introduced into *Arabidopsis thaliana* (ecotype Columbia) wild-type by floral dip method for stable expression [22]. The Arabidopsis transformants were first selected by hygromycin (30 mg/L), and then confirmed by PCR of genomic DNA using primers EgWRI1-pro F/R as described above. PCR was carried out using Taq DNA polymerase (TransGen Biotechnology, Beijing) under the following thermal program: an initial denaturation at 94°C for 5 min followed by 30 cycles of 94°C for 10 s, 53°C for 15 s, and 72°C for 10 s, with a final extension at 72°C for 5 min. PCR products were checked by electrophoresis on a 1% agarose gel.

### Abiotic stress and hormone treatments

To explore the expression pattern of *EgWRI1* promoter, ten-day-old seedlings of the T1 generation of transgenic Arabidopsis harboring ProEgWRI1::GUS were subjected to various stress treatments. For ethylene treatment, transgenic plants were sprayed with 80 μM ethephon solution once every 12 h for 96 h. For low temperature and light treatments, transgenic plants were planted at 15°C and in dark, respectively, with other conditions are the same with the control (as mentioned in Materials). Quantitative analysis of GUS activity in each plant was carried out after treatments for12/24h, 48h, and probably 96h.

### Gus histochemistry

Histochemical GUS staining was performed on seedlings and various tissues of Arabidopsis as described by Jefferson *et al*. [23]. The plant tissues were collected from both untransformed and transformed plants at approximately the same position. For the assay, the selected seedlings and tissues were incubated in GUS staining buffer (2 mM 5-bromo-4-chloro-3-indoyl glucuronide (X-Gluc), 5 mM K3Fe(CN)6, 100 mM Na3PO4 (pH 7.0), 5 mM K4Fe(CN)6, 0.2% Triton X-100 and 10 mM EDTA) overnight at 37°C in dark. The tissues were first decolorized with 70% ethanol, and then imaged by stereomicroscopy. Quantitative analysis of GUS activity in transgenic Arabidopsis was determined according to Jefferson *et al*. [23]. The protein concentrations of plant extracts were measured as descried by Bradford [24]. GUS activity was calculated as pmol 4-Methylumbelliferone (4-MU) per min per microgram protein. Three replicates were performed for each sample.

## Results

### Isolation and sequence analysis of the EgWRI1 promoter

**Fig 1:**
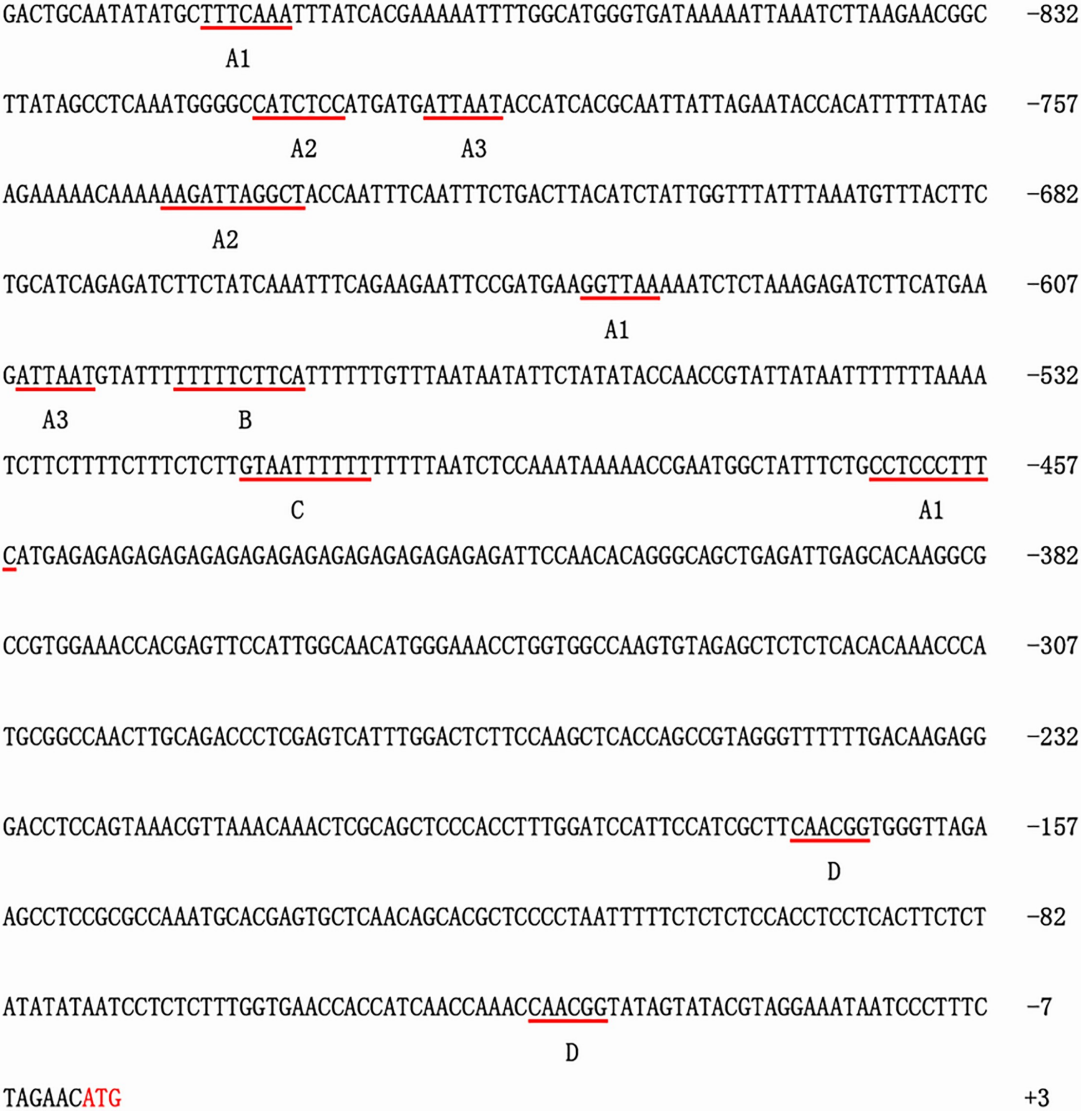
Nucleotide sequence of the oil palm *EgWRI1* promoter (906bp). The transcription start site “ATG” was colored in red. Putative regulatory elements were highlighted by red line, and labeled by different capital letters (A-D). A1: light responsive element; A2: part of light-responsive; A3: part of a conserved DNA module involved in light responsiveness; B: cis-acting element involved in defense and stress responsiveness; C: heat stress-responsive; D: MYBHv1 binding site.

According to *EgWRI1* cDNA (accession number: XM_010924626), the 906 bp upstream promoter region of *EgWRI1* was predicted in the *Elaeis guineensis* genomic sequence (taxid: 51953) and was isolated from genomic DNA by PCR using the primers EgWRI1-pro F/R. Then, to detect the putative functional elements, the sequence of the *EgWRI1* promoter was analyzed by the PlantCARE database [20]. The results revealed that various putative plant regulatory elements are present in the *EgWRI1* promoter region (Fig 1). There were seven elements involved in light responsiveness, including A1 (TTTCAAA), A2 (CATCTCC), and A3 (ATTAAT) (Fig 1). Other elements were also identified in the *EgWRI1* promoter region, such as a defense and stress-responsive element (TTTTTCTTCA; the motif labeled by B), a heat-responsive element (GTAATTTTTT; the motif labeled by C),(Fig 1).

### Expression patterns mediated by the EgWRI1 promoter in transgenic Arabidopsis

**Fig 2:**
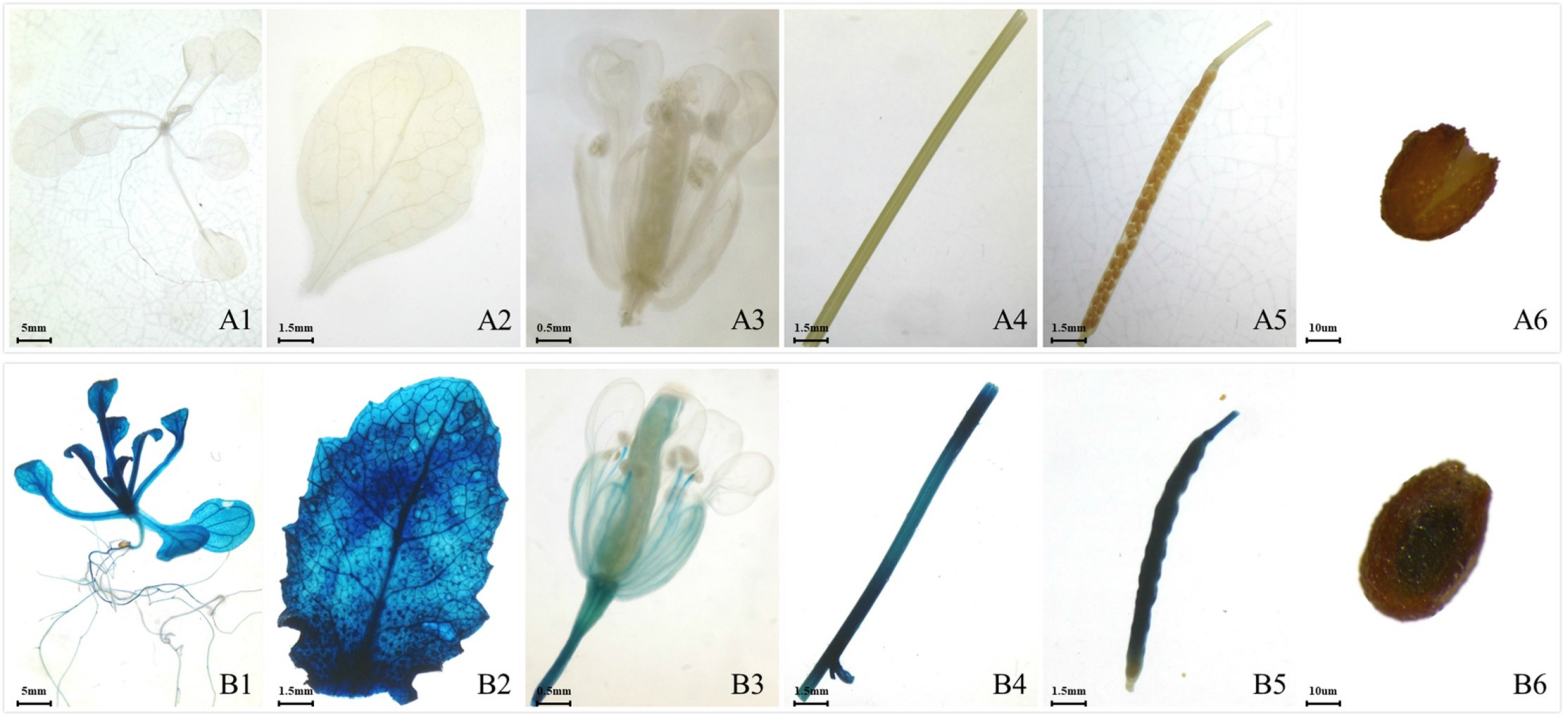
Histochemical GUS staining of transgenic Arabidopsis harboring the EgWRI1-pro-GUS fusion. The results from transgenic Arabidopsis: B1-B6; the untransformed Arabidopsis were used as the negative control: A1-A6. 1: three-week-old seedling; 2: leaves; 3: flowers; 4: stems; 5: silique coats; 6: seeds.

To test the promoter activity, the construct ProEgWRI1::GUS was transformed into Arabidopsis. The positively transformed plants were first selected by hygromycin and then confirmed by PCR (data not shown). The T1 generation of transgenic Arabidopsis was subjected to a GUS histochemical assay in various tissues: seedlings, leaves, flowers, stems, silique coats, and seeds. The untransformed wild-type plants were used as the negative control. The results showed that GUS staining was very strong in whole three-week-old seedlings, especially in the stems and leaves (Fig 2B1, 2B2 and 2B4). No GUS staining was detected in the negative control (Fig 2A1–2A6). In male flowers, the filament was also stained, but the anther was not (Fig 2B3). There was very slight GUS staining detected on the surface of the pistil (Fig 2B3). Moreover, GUS staining was strong in the silique coats but weak in the seeds (Fig 2B5 and 2B6).

### The activity of the EgWRI1 promoter in response to treatment with different growth environments

**Fig 3:**
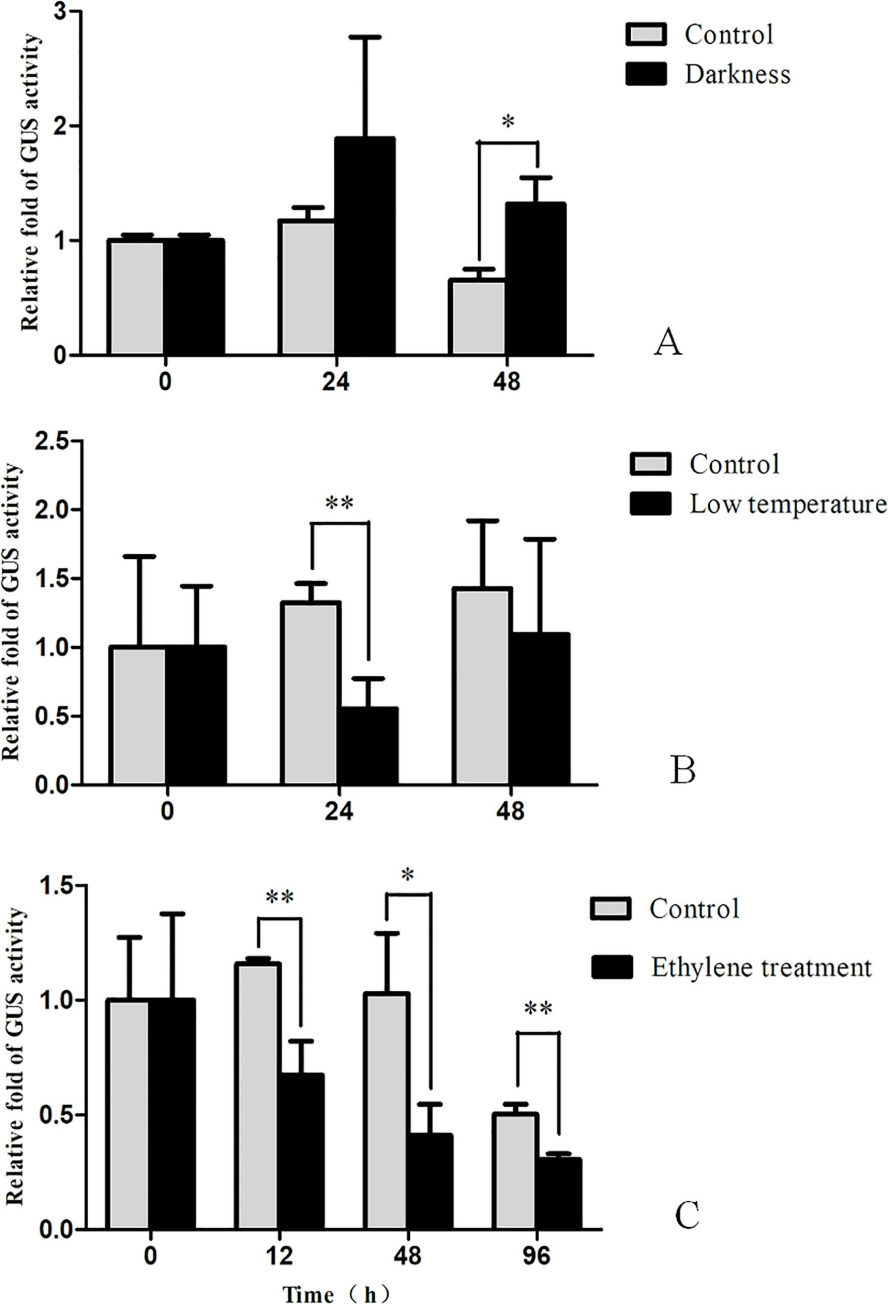
Quantification of GUS activity in leaves of transgenic Arabidopsis harboring the ProEgWRI1::GUS fusion under the treatment of darkness (A), low temperature (B; 15°C), and ethylene (C), respectively.

Since the *EgWRI1* promoter contained some putative stress-responsive elements, further work was carried out to investigate whether it was induced under various stress conditions. Ten-day-old seedlings of the T1 generation were treated with darkness, low temperature (15°C), and exogenous ethylene, and the other conditions were the same as the control conditions. The quantification of GUS activity in the transformed plants from both treatments and controls was performed. As shown in Fig 3A, the GUS activity of transgenic plants treated with darkness did not show an obvious change after 24 hours but exhibited a significant increase by 1-fold after 48 hours when compared with that of the control. However, the GUS activity of transgenic plants under low-temperature treatment displayed the opposite result and decreased significantly after 24 hours but recovered after 48 hours when compared with that of the control (Fig 3B). In addition, exogenous ethylene significantly reduced GUS activity (approximately 40–60%) in transgenic plants compared with the control (Fig 3C) (S1 Table). These results indicated that the activity of the *EgWRI1* promoter was induced or suppressed in a severe stress environment.

## Discussion

In the present study, we characterized the promoter of *EgWRI1* from oil palm by determining the expression patterns and the response to environmental stress in transgenic Arabidopsis. The *EgWRI1* promoter was first isolated and fused to a promoterless GUS reporter gene, yielding the ProEgWRI1::GUS construct. Then, the construct was transformed into Arabidopsis to identify the activity of the *EgWRI1* promoter by GUS histochemical assay. The results indicated that GUS staining was very strong in whole seedlings (Fig 2B1), especially the stems, leaves, and siliques (Fig 2). The transcript level of fruit-specific *EgWRI1* has only been detected in oil palm fruit, which increased by 7.5-fold in the mesocarp during ripening [3]. Moreover, *EgWRI1* showed an even higher expression level in the endosperm than in the mesocarp [18]. Unfortunately, no transcript data of *EgWRI1* in other oil palm tissues (such as leaves, flowers, and roots) have been reported. Whether the *EgWRI1* promoter functions exactly the same in transgenic Arabidopsis as it does in oil palm remains unclear. The expression pattern of *AtWRI1* in Arabidopsis indicated that *WRI1* is ubiquitously expressed in diverse tissues, including seedlings, roots, shoots, leaves, stems, flowers, and siliques [6]. The *wri1* mutant showed reduced primary root growth [25], which is consistent with the activity of the *EgWRI1* promoter. Therefore, the *EgWRI1* promoter exhibited similar activity to the original *AtWRI1* promoter, with even stronger efficiency. Our results also indicated that EgWRI1 may play important roles in various tissues for vegetative development and flowering. However, GUS staining was strong in the silique coats and weak in the seeds from ProEgWRI1::GUS transgenic Arabidopsis (Fig 2B6), which might be caused by the particular activity of the fruit-specific *WRI1* promoter.

The bioinformatic analysis of the *EgWRI1* promoter revealed several putative plant regulatory elements, such as light-responsive elements, defense and stress-responsive elements, and heat stress-responsive elements (Fig 1). To verify whether the *EgWRI1* promoter was affected by environmental stress, the GUS activity of leaves from transgenic Arabidopsis under low-temperature and darkness treatments were quantified. The results indicated that GUS activity increased one-fold after 48 hours of darkness treatment (Fig 3A). Therefore, darkness induced the expression of *EgWRI1* to adjust to the change in the growth environment. One hypothesis is that the lack of photosynthesis results in a deficiency of chemical energy converted from light energy in plants, which is used for metabolism, growth, and energy storage [1]. To maintain plant homeostasis, the storage carbohydrates fuel additional starch, lipid, and amino acid synthesis in the dark. Moreover, it has been reported that BnWRI1 from *Brassica napus* decreased storage carbohydrates and increased soluble sugars to facilitate the carbon flux to lipid anabolism [26]. Thus, the activity of the *EgWRI1* promoter would be enhanced to induce the expression of *WRI1* to increase carbon flux for plant growth when treated with darkness. However, the related mechanisms need to be elucidated.

On the other hand, the GUS activity of transgenic plants under low-temperature treatment was significantly reduced but recovered after 48 hours (Fig 3B). It is known that polyunsaturated membranes are essential for maintaining cellular functions and plant viability at low temperatures [27]. Additionally, the low-temperature treatment could induce the expression of chloroplastic *FAD8* to maintain the fluidity of membranes [28]. WRI1 plays important roles in oil synthesis and accumulation [6]. To adjust to low temperatures, transgenic Arabidopsis might need to decrease the activity of EgWRI1 to limit the carbon flux into oil to synthesize constitutive membrane lipids. This hypothesis also needs to be verified with further experiments.

Given that WRI1 tightly correlates with oil accumulation and seed/fruit maturity, we also tested whether ethylene affected the activity of the *EgWRI1* promoter in transgenic Arabidopsis. The results indicated that exogenous ethylene reduced GUS activity by 40–60% (Fig 3C). It has been reported that plant growth is reduced by exposure to ethylene [29]. Thus, it is reasonable that ethylene regulates the expression of *EgWRI1*, which is an important transcription factor involved in accelerating vegetative development in overexpression Arabidopsis [17]. The phytohormone ethylene affects a variety of basic plant processes throughout the whole life cycle of plants, ranging from growth and development to stress responses [30]. However, the mechanism by which ethylene regulates the expression of *WRI1* remains unknown.

In summary, these results reveal the activity of the *EgWRI1* promoter from oil palm in transgenic Arabidopsis. The expression pattern mediated by the *EgWRI1* promoter in transgenic Arabidopsis has been tested. The results showed that it exhibited very strong efficiency in whole seedlings, which is similar to the original AtWRI1 promoter, indicating an even stronger function. Furthermore, we also detected the influence of *EgWRI1* promoter activity in response to changes in the growth environment, and the results indicated that the activity of the *EgWRI1* promoter was induced under darkness but suppressed significantly when exposed to exogenous ethylene. When treated with low temperature, the activity of the *EgWRI1* promoter was first reduced after 24 hours but recovered after 48 hours. We also noticed that the *EgWRI1* promoter contains an MYBHv1-binding site (Fig 1). The MYB family of proteins is large and functionally diverse in all eukaryotes, and most of them function as transcription factors [31]. Therefore, this result indicates that the upstream regulator of fruit-specific *EgWRI1* may derive from proteins of the MYB family. Overall, these results will be helpful for understanding the function of the *EgWRI1* promoter in palm plants.

## Supporting information

S1 Table Quantification of GUS activity under the treatment of darkness, low temperature and ethylene. (XLSX)
